# Widely Targeted Metabolomics Provides New Insights into Nutritional Profiling and Reveals the Flavonoid Pathway of Pea (*Pisum sativum* L.)

**DOI:** 10.3390/foods13131970

**Published:** 2024-06-21

**Authors:** Longqing Sun, Li Li, Hongwei Chen, Xuesong Han, Liangjun Liu, Changyan Liu

**Affiliations:** Hubei Key Laboratory of Food Crop Germplasm and Genetic Improvement, Food Crops Institute, Hubei Academy of Agricultural Sciences, Wuhan 430064, China; lqsun@hbaas.com (L.S.);

**Keywords:** pea, metabolomics, nutritional, flavonoids, isoflavones

## Abstract

To learn more about the nutritional composition and health benefits for human consumers of peas, we used a widely targeted metabolomics-based approach to reveal the metabolite components from three main varieties, and a total of 1095 metabolites were identified. A comparison of 487 differentially accumulated metabolites shared among three varieties of fresh and dried peas found most of the amino acids and derivatives were downregulated and most of the lipids and flavonoids were upregulated in dried peas. Furthermore, comparing the main nutrient profiles exclusively showed that there were few differences in free fatty acids, sugars, vitamins, and alkaloids between dried and fresh peas. Peas are especially enriched with B-group vitamins. Through detailed identification and classification, the flavonoid pathway of peas was revealed; a variety of glycosylated derivatives from kaempferol, quercetin, and luteolin were confirmed to be abundant in peas. It was also found that isoflavones are richer in peas than in many other plants, and putatively the isoflavone synthesis pathway originates from liquiritigenin and naringenin. Our study not only offers guidance for understanding the nutritional components of peas, but also provides the basis for healthy diet analysis of the edible value and health benefits of peas.

## 1. Introduction

Pea (*Pisum sativum*) is one of the most important cultivated crops in the world, and its seeds, tender pods, and young seedlings can be eaten. The green pea not only can be eaten fresh, but also can be dehydrated and quick-frozen or processed and stored in cans. The seed flour of dried peas is used as an additive in breads, cakes, and nutritional foods, etc. Recently, people have paid more and more attention to improving the nutritional and medical value of crops [1]. Due to the abundance of nutritional and bioactive ingredients in peas, their consumption has been associated with a wide range of health benefits [2]. Therefore, a full understanding of their different nutritional components is the basis needed to help people meet their nutritional requirements and develop the pea as a functional crop.

Previous studies suggested that pea was rich in diverse metabolic components. Headspace solid-phase microextraction gas chromatography–mass spectrometry combined with untargeted metabolomic approaches differentiated the aromatic profiles of seven yellow pea flours [3]. In order to study the in vivo antioxidant activities of green pea hull (GPH), a total of 31 phenolics, comprising four phenolic acids, 24 flavonoids, and three other phenolics, were tentatively identified using ultra-high-pressure liquid chromatography with Orbitrap mass spectrometry [4]. In another study, 42 metabolites detected from yellow pea hull by UHPLC-LTQ-Orbitrap-MS showed a positive effect on SOD, GSH-Px, MDA, and T-AOC using the D-gal model in rats [5]. Analysis of separated seed coats in pea by LC/ESI-MS revealed significantly higher contents of proanthocyanidins, quercetin, myricetin rhamnosidase, and hydroxylated fatty acids in dormant compared to non-dormant genotypes [6]. Metabolite profiling of seeds by gas chromatography–mass spectroscopy revealed the role of albumin 2 in regulating polyamine metabolism in developing pea seeds [7]. Rhizobium inoculation induced the change of triterpenoid soyasapogenol in seed, which led to peas’ increasing resistance level against *Didymella pinodes* [8]. Sakuranetin, the flavonoid phytoalexin which can be induced by UV radiation, was detected in peas by metabolomics [9]. The optimized method using gas chromatography coupled with mass spectrometry was shown to be reliable for the assessment of the organ and species metabolic profiles of pea roots and leaves [10]. In order to understand the mechanism regulating pea pod coloration, 34 differential metabolites related to the flavonoid metabolism pathway were identified from two pea accessions with green pods and yellow pods using metabolome analysis [11]. In addition, a systematic evaluation of the performance of direct introduction ESI and MALDI coupled with FTICR MS was applied to non-targeted metabolomics of pea root exudates [12]. Another work found that common changes were induced by different continuous cropping treatments in flavonoid, glutathione, and linoleic acid metabolism in pea roots [13]. Comprehensive analysis of the omics data also revealed that the flavonoid and isoflavonoid biosynthesis pathways play key roles in the response of pea roots to continuous cropping obstacles [14]. Moreover, 41 polar metabolites were characterized by the GC-FID method in the shoot tip, stem, stipules, and tendrils of a semi-leafless pea under progressive soil drought and subsequent re-watering [15]. The application of gas chromatography coupled with a flame ionization detector, as well as gas chromatography coupled with mass spectrometry, identified 51 polar metabolites during the seed germination and early development of peas [16]. Although these previous studies were relatively in-depth, there are few reports on the systematic analysis of nutritional metabolites in peas. The metabolic components in fresh peas and dried peas are still unclear. Furthermore, data on the breeding of new varieties to improve the nutritional quality of peas are still quite limited. Therefore, more work needs to be carried out to provide a foundation for study of the health benefits and nutritional value of peas.

Metabolic profiling based on liquid chromatography–tandem mass spectrometry (LC-MS/MS) is currently very popular in the study of plant metabolomics. Widely targeted metabolomics has significant advantages for large-scale detection, identification, and quantification of metabolites and their accumulation patterns in plants, allowing identification of more than 1000 compounds for a single detection. Compared with other metabolite detection methods, it has the characteristics of high throughput, high sensitivity, and high repeatability. It also plays an important role in the identification of various food nutritional metabolites. More than 260 nutrients were identified from crops (wheat, rice, and corn) and fruits (grape, banana, and mango) using LC-MS-based non-targeted and targeted metabolome analyses [17]. Metabolomic variations were investigated among six important species of *Poaceae* with a total of 17 cultivars, including wheat, maize, rice, sorghum, foxtail millet, and broomcorn millet based on the UPLC-ESI-MS/MS system [1]. Analysis of the accumulation patterns of common nutritional metabolites among six coarse cereals found that the accumulation of carbohydrates follows a conserved pattern [18].

In this study, the metabolites of fresh and dried seeds of three pea varieties were analyzed and compared by widely targeted metabolomics analysis based on the UPLC-ESI-MS/MS system to reveal the differences between them. Specifically, the accumulation of nutrient compositions and bioactive constituents in fresh and dried peas were systematically evaluated based on classification and comparison. Moreover, the metabolic pathway of flavonoids in peas was reconstructed using the Kyoto Encyclopedia of Genes and Genomes (KEGG) platform as a reference database. The results of our study not only provide a reference for healthy diets including peas, but also give valuable clues for the targeted use of peas.

## 2. Materials and Methods

### 2.1. Plant Materials

To study the metabolites in peas, the three main cultivation varieties planted in Hubei province, Zhongwan 4 (ZW4), Zhongwan 6 (ZW6) and Changshouren (CSR), were selected for analysis. ZW4 and ZW6 were bred by the Institute of Animal Husbandry, Chinese Academy of Agricultural Sciences. ZW4 is one of the parents of ZW6 (Figure S1). CSR was bred by Taiwan Xingnong Seedling Co., Ltd. The three varieties were sown under autumn conditions in an experimental field at the Food Crops Institute, Hubei Academy of Agricultural Sciences, Wuhan, Hubei, China. The sowing date was 30 October 2021. Each variety of pea was planted in three plots as 3 replications. Each plot was 2 m long and 1.5 wide. Each variety was planted in three rows, with 50 cm between rows and 10 cm between plants. The experiment was irrigated and kept pest- and disease-free using normal agronomic practice. When the peas went into the full bloom stage, hang tags were used to mark the blooming flowers. After 20 days, 10 fresh pods with hang tags were picked from each variety in each plot. After peeling the fresh pod, one seed was removed with tweezers. Then, 10 seeds of the same size were obtained from 10 fresh pods as one replicate. Three biological replicates were obtained from three plots, respectively. During the harvest period, the sampling method for dry seeds was the same as that used for fresh seeds. The fresh and dry seeds of each variety were sampled and immediately frozen in liquid nitrogen. The frozen samples were stored at −80 °C in a freezer for subsequent metabolome analysis.

### 2.2. Metabolite Sample Preparation

The fresh seeds and dry seeds from ZW4, ZW6, and CSR, including nine fresh pea samples ZW4F, ZW6F, CSRF, and nine dried pea samples ZW4D, ZW6D, CSRD, were freeze-dried by vacuum freeze-dryer (Scientz-100F, Ningbo Xinzhi Biotechnology Co., Ltd., Ningbo, China). The freeze-dried samples were crushed using a mixer mill (MM 400, Retsch GmbH, Haan, Germany) with a zirconia bead for 1.5 min at 30 Hz. Dissolve 50 mg of lyophilized powder with 1.2 mL 70% methanol solution, vortex 30 s every 30 min for 6 times in total. Following centrifugation at 12,000 rpm for 3 min, the extracts were filtrated before UPLC-MS/MS analysis. Our final dataset comprises the metabolomics data from 18 samples (fresh peas or dried peas, three varieties, three biological replicates).

### 2.3. UPLC Conditions

The sample extracts were analyzed using an UPLC-ESI-MS/MS system (Metware Biotechnology Co., Ltd., Wuhan, China). The analytical conditions were as follows, UPLC: column, Agilent SB-C18 (1.8 µm, 2.1 mm * 100 mm). The mobile phase consisted of solvent A (pure water with 0.1% formic acid) and solvent B (acetonitrile with 0.1% formic acid). Sample measurements were performed with a gradient program that employed the starting conditions of 95% A, 5% B. Within 9 min, a linear gradient to 5% A, 95% B was programmed, and a composition of 5% A, 95% B was kept for 1 min. Subsequently, a composition of 95% A, 5.0% B was adjusted within 1.1 min and kept for 2.9 min. The flow velocity was set as 0.35 mL per minute; The column oven was set to 40 °C. The injection volume was 2 μL. The effluent was alternatively connected to an ESI-triple quadrupole-linear ion trap (QTRAP)-MS.

### 2.4. ESI-Q TRAP-MS/MS

The ESI source operation parameters were as follows: source temperature 500 °C; ion spray voltage (IS) 5500 V (positive ion mode)/-4500 V (negative ion mode); ion source gas I (GSI), gas II (GSII); curtain gas (CUR) was set at 50, 60, and 25 psi, respectively; the collision-activated dissociation (CAD) was high. QQQ scans were acquired as MRM experiments with collision gas (nitrogen) set to medium. DP (declustering potential) and CE (collision energy) for individual MRM transitions were applied with further DP and CE optimization. A specific set of MRM transitions were monitored for each period according to the metabolites eluted within this period.

### 2.5. Identification of Metabolites

Analyst 1.6.1 software was employed to analyze the mass spectrometry data. To produce a matrix containing less biased and redundant data, peaks were manually checked for signal/noise (S/N) > 10. Then, we removed the redundant signals caused by different isotopes, in-source fragmentation, K+, Na+, and NH4+ adducts, and dimerization. Metabolites in the samples were identified by comparing accurate m/z values, retention times (RT), and fragmentation patterns in the self-built Metware Database (MWDB) and public databases (MassBank and METLIN).

### 2.6. Statistical Analysis

Unsupervised principal component analysis (PCA) was performed using the statistics function prcomp within R (http://www.r-project.org (accessed on 20 August 2022)). The data were unit variance scaled before unsupervised PCA. The hierarchical cluster analysis (HCA) results for samples and metabolites were presented as heatmaps with dendrograms. The Pearson correlation coefficients (PCCs) between samples were calculated by the cor function. Both HCA and PCA were carried out with the R package Complex-Heatmap. 

### 2.7. Differential Metabolites Selected

For two-group analysis, the differential metabolites were determined by VIP (VIP ≥ 1) and absolute Log2 FC (|Log2 FC| ≥ 1.0). VIP values were extracted from OPLS-DA results, which also contained score plots and per-(|Log2 FC| ≥ 1.0), and per-mutation plots were generated using the R package MetaboAnalystR (http://www.r-project.org (accessed on 20 August 2022)). The data were log2 transformed and mean centered before OPLS-DA. In order to avoid overfitting, a permutation test (200 permutations) was performed. 

### 2.8. KEGG Annotation and Enrichment Analysis

Identified metabolites were annotated using the KEGG Compound database (http://www.kegg.jp/kegg/compound/ (accessed on 1 September 2022)); annotated metabolites were then mapped to the KEGG Pathway database (http://www.kegg.jp/kegg/pathway.html (accessed on 1 September 2022)). Pathways mapped to significantly regulated metabolites were then fed into Metabolite Sets Enrichment Analysis (MSEA), and their significance was determined by hypergeometric test’s *p*-values.

## 3. Results and Discussion

### 3.1. Metabolite Profiles of Different Peas

**Figure 1:**
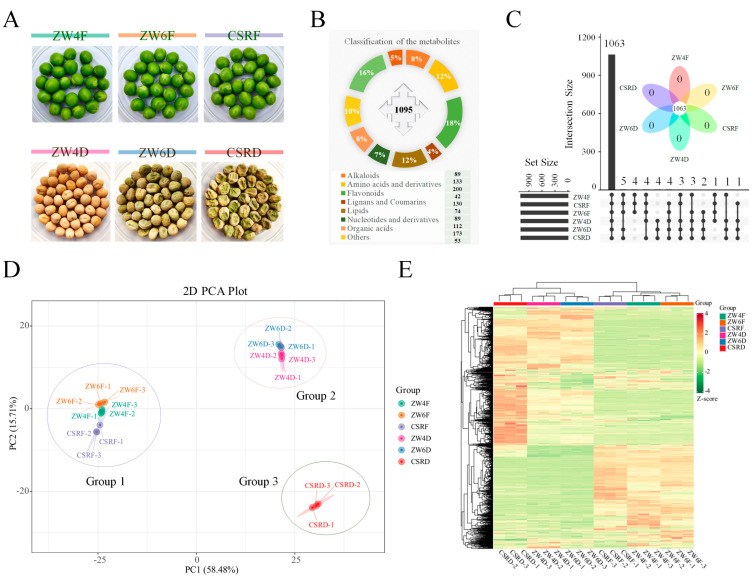
Metabolite profiles of fresh peas and dried peas. (**A**) The phenotypic traits of fresh peas at full grain-filling stage and dried peas at mature stage from three different varieties. (**B**) Venn diagram analysis shows the overlapping and unique metabolites among different samples and an upset plot of the number of metabolites detected in fresh peas and dried peas from different varieties. (**C**) Classification of the 1095 metabolites from fresh peas and dried peas. (**D**) Principal component analysis of metabolites in different peas. (**E**) The cluster heatmap of all metabolites from different peas. Each metabolite is represented by a row, and each sample is represented by a column.

In China, the fresh pea is a popular vegetable, and the dried pea is known as a traditional miscellaneous grain crop. For better developing the pea as functional crop, we carried out a wide identification of the metabolites in pea seeds by the widely targeted metabolomics approach to exploit and utilize the food value and health function of peas. Three main varieties, ZW4, ZW6 and CSR, were selected in this study. The fresh pea of ZW4 is green, but the dried pea is yellow-white. ZW6 and CSR have a similar grain appearance (Figure 1A). A total of 1095 different annotated metabolites were identified among different peas, including 89 alkaloids, 133 amino acids and derivatives, 200 flavonoids, 42 lignans and coumarins, 130 lipids, 74 nucleotides and derivatives, 89 organic acids, 173 phenolic acids, 53 terpenoids, and 112 other compounds (Figure 1B). There were 1063 metabolites shared among all tested peas. Four metabolites were specifically found in fresh peas, and another four were specifically found in dried peas. However, no specific metabolites were found in any varieties (Figure 1C). PCA was performed on the identified metabolites to explore the diversity of metabolites among the metabolite profiles of different samples. According to the PCA, PC1 and PC2 represented a 58.48% and 15.71% contribution to the differences among different peas, respectively. Meanwhile, the fresh peas and dried peas were separated in PC1. The dried pea samples of CSR were separated from other peas in PC2. The samples were obviously divided into three different groups, indicating that the samples in each group had the same metabolic profiles. Group1 contained all fresh pea samples. Group2 included dried pea samples of ZW4 and ZW6; Group3 included dried pea samples of CSR (Figure 1D). This indicated that the metabolite profiles from different varieties of fresh peas were similar, and the genetically related varieties were more similar with each other in metabolic profiles even if there were differences in appearance. Overall, there were significant differences in accumulation patterns between fresh and dry seeds, which were supported by the hierarchical cluster analysis (HCA) (Figure 1E). In addition, it was also found that the correlation between replicates was greater than 0.98 by correlation analysis (Figure S2), indicating the high reproducibility of the metabolome data.

### 3.2. Identification of the Differentially Accumulated Metabolites (DAMs)

**Figure 2:**
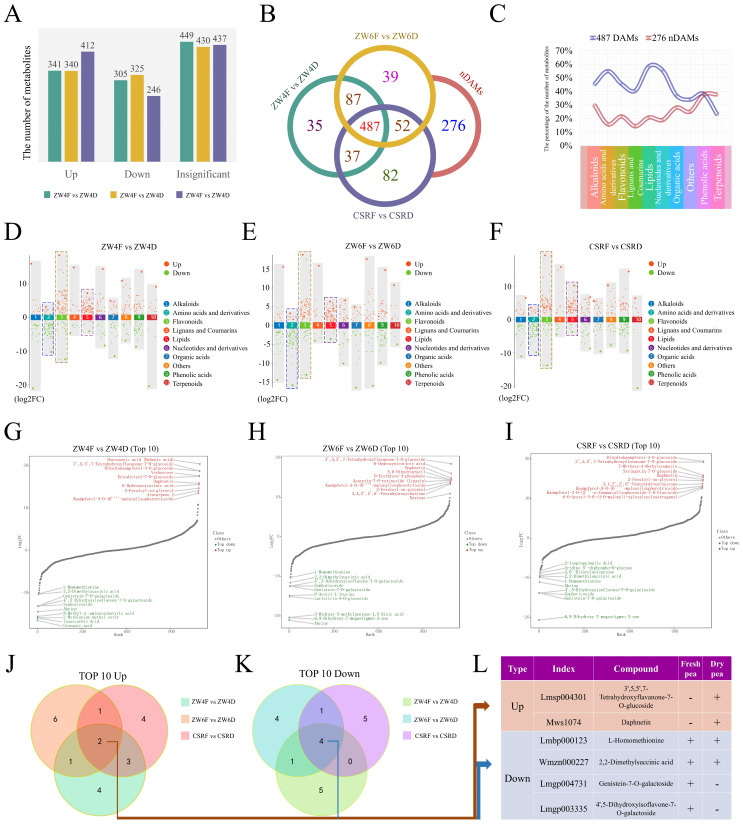
Identification of differential metabolites for three comparison groups, ZW4F vs. ZW4D, ZW6F vs. ZW6D, and CSRF vs. CSRD. (**A**) The number of significantly upregulated and downregulated metabolites from different comparison groups. (**B**) Venn diagram shows the overlapped and sample-specific differential metabolites. (**C**) The percentage of 487 DAMs and 276 nDAMs after divided into 10 different categories. (**D**) The 487 DAMs were divided into 10 categories for ZW4F vs. ZW4D, the red dots represent upregulated metabolites, the green dots represent downregulated metabolites. (**E**) The 487 DAMs were divided into 10 categories for ZW6F vs. ZW6D, the red dots represent upregulated metabolites, the green dots represent downregulated metabolites. (**F**) The 487 DAMs were divided into 10 categories for CSRF vs. CSRD, the red dots represent upregulated metabolites, the green dots represent downregulated metabolites. (**G**) The volcano plot of the top 10 upregulated and downregulated metabolites for ZW4F vs. ZW4D. (**H**) The volcano plot of the top 10 upregulated and downregulated metabolites for ZW6F vs. ZW6D. (**I**) The volcano plot of the top 10 upregulated and downregulated metabolites for CSRF vs. CSRD. (**J**) Venn diagram analysis shows the overlapping and unique metabolites among different comparison groups for top 10 upregulated metabolites. (**K**) Venn diagram analysis shows the overlapping and unique metabolites among different comparison groups for top 10 downregulated metabolites. (**L**) The distribution for the upregulated and downregulated metabolites from the Venn analysis in dried and fresh peas.

To examine the difference between dried and fresh peas of the same variety, we used VIP (VIP ≥ 1) and absolute Log_2_ FC (|Log_2_ FC| ≥ 1.0) to select significantly differential metabolites. A total of 646 DAMs (341 upregulated, 305 downregulated) were identified in the comparison of ZW4F vs. ZW4D, 665 DAMs (340 upregulated, 325 downregulated) were identified in the comparison of ZW6F vs. ZW6D, and 658 DAMs (412 upregulated, 246 downregulated) were identified in the comparison of CSRF vs. CSRD (Figure 2A, Figure S3). Detailed information on the DAMs is listed in Table S1. A total of 487 DAMs were shared by all three comparisons, and 276 metabolites showed no significant differences among the three comparisons. Totals of 35, 39 and 82 differential metabolites were specifically detected in ZW4F vs. ZW4D, ZW6F vs. ZW6D, and CSRF vs. CSRD, respectively. It indicated that more differences were present in the comparison of CSRF vs. CSRD (Figure 2B). Furthermore, many specifically differential metabolites were detected from the subclasses of free fatty acids, organic acids, and phenolic acids in CSRF vs. CSRD (Table S2). The relative proportions of amino acid and derivatives, lipids, nucleotides and derivatives in the group of 487 DAMs were high, all nearly 60%. The percentages of phenolic acids and terpenoids were higher than those of the other categories in the group of 276 non-differentially accumulated metabolites (nDAMs), both of which were close to 40% (Figure 2C). In addition, after comparing the differences between fresh and dried peas in the three comparisons respectively, it was found that amino acids and derivatives, flavonoids and lipids showed great changes. That is, most of the amino acids and derivatives were downregulated in dried peas; however, lipids and flavonoids were upregulated (Figure 2D–F). Although most of the amino acids and derivatives were downregulated in all three comparisons, the following metabolites were upregulated: N-palmitoylglycine, y-Glu-Cys, N-acetyl-L-tryptophan, L-glycine, N-acetyl-L-phenylalanine, Cyclo(Ala-Glv), N,N’-dimethylarzinine, NG,NG-dimethyl-L-arginine, N-acetvl-L-glutamine, L-glycyl-L-proline, N6-acetyl-L-lysine, homoarzinine, and trimethyllysine (Figure S4A). Within the category of lipids, all metabolites of LPC and LPE subclasses were upregulated in three comparisons, of which LysoPC (18:1), LysoPC (18:1) (2n isomer), LysoPE (18:0) (2n isomer), LysoPE (18:1), and LysoPE (18:1) (2n isomer) were changed greatly (Figure S4B). In the categories of terpenoids, lignans and coumarins, only a few compounds were changed in three comparisons (Figure 2D–F). 

For obtaining metabolites with extreme variation in different comparison groups, we identified the top 10 compounds by using Log_2_FC. Venn diagram analysis showed the overlapping and unique metabolites by using the top 10 upregulated or downregulated metabolites among different comparison groups (Figure 2G–I). Two upregulated and four downregulated compounds were found in all comparison groups (Figure 2J,K). They were 3′,5,5′,7-tetrahydroxyflavanone-7-O-glucoside, daphnetin, L-homomethionine, 2,2-dimethylsuccinic acid, genistein-7-O-galactoside, and 4′,5-dihydroxyisoflavone-7-O-galactoside, respectively, of which 3′,5′,5′,7-tetrahydroxyflavanone-7-O-glucoside and daphnetin were not detected in the fresh peas. Genistein-7-O-galactoside and 4′,5-dihydroxyisoflavone-7-O-galactoside were not detected in dried peas (Figure 2L).

Furtherly, in order to understand the main biochemical metabolic pathways, the differential accumulated metabolites from each comparison group was annotated by the KEGG database (Table S3). After comparing the top 20 KEGG pathways from each comparison group, the results indicated that the pathways enriched among different comparison groups were similar. Meanwhile, biosynthesis of cofactors and biosynthesis of amino acids were the two highest enrichment pathways in all three comparison groups. However, it uniquely affects glycine, serine and threonine metabolism, alanine, aspartate and glutamate metabolism in the comparison of ZW4F vs. ZW4D. Linoleic acid and phenylalanine pathways were uniquely affected in the comparison of CSRF vs. CSRD (Figure S5).

### 3.3. Comparative Analysis of the Important Nutrients and Anti-Nutrients in Peas

The WHO estimated that almost 2 billion people around the world are at risk of micronutrient deficiencies [19]. Therefore, a deep understanding of the nutrients in crops is essential for health. Metabolomics has been efficiently used to analyze the type, content and accumulation pattern of metabolites [20], and also can be utilized in the analysis of nutritional compounds for many plants [21]. For example, a widely targeted metabolic profiling analysis was performed to investigate nutritional diversity among six coarse cereals including millet, grain sorghum, oat, coix, buckwheat and quinoa [18]. In another work, the widely targeted metabolomics-based approach was used to reveal the nutritional components of black, red, glutinous, and common white rice [22]. Another study used LC-MS-based non-targeted metabolomics analysis to understand the nutrient composition of 10 kinds of fruits [23]. In the current century, the development of medicine and molecular biotechnology has led to the identification of the important roles of nutrients in the protection of human health and in the prevention of human diseases [24]. In addition, studies of metabolic profiles in crops and fruits discovered the complementary patterns of essential nutrients and provided metabolomic evidence for a healthy diet [13]. As an important food and vegetable crop, the application of peas in food, medicine, and industry has been gradually developed [25]. In order to better develop their edible value and provide a reference for peas for healthy diets, we performed widely targeted metabolic profiling analyses of fresh peas and dried peas with UPLC-ESI-MS/MS. Therefore, the main nutrients and anti-nutrients were identified for revealing the nutritional profiles of peas.

**Figure 3:**
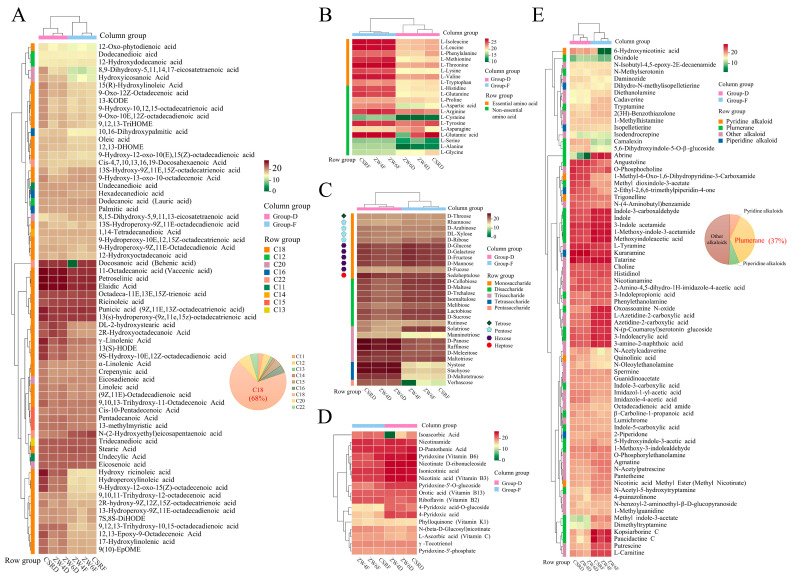
Profiles of the nutrients and anti-nutrients in fresh peas and dried peas from three varieties. (**A**) Heatmap of free fatty acids in fresh peas and dried peas. (**B**) Heatmap of amino acids in fresh peas and dried peas. (**C**) Heatmap of sugars in fresh peas and dried peas. (**D**) Heatmap of vitamins in fresh peas and dried peas. (**E**) Heatmap of alkaloids in fresh peas and dried peas. The mean content of each metabolite from three biological replicates was normalized by log2 transformation. Columns represent the different peas, and metabolites are represented by rows. Group-F represents the group of fresh peas, and Group-D represents the group of dried peas.

Free fatty acids, as a class of important substances for storing energy and providing fuel essential for cellular survival [26], play an important role in human metabolism. The free fatty acid metabolome profiles show that peas are rich in long-chain fatty acids from C12 to C18 except C17, and that C18 fatty acids account for up to 68% of the total free fatty acids. Two C11 medium chain fatty acids, undecanedioic acid and undecylic acid, were identified. In addition, peas also contain C20 and C22 types of ultra-long chain fatty acids, including 8,15-dihydroxy-5,9,11,13-eicosatetraenoic acid, 8,9-Dihydroxy-5,11,14,17-eicosatetraenoic acid, eicosadienoic acid, Ethyl 9-Hydroxy-10,12-octadecadienoic acid, eicosenoic acid, hydroxyicosanoic acid, cis-4,7,10,13,16,19-docosahexaenoic acid, N-(2-hydroxyethyl) eicosapentaenoic acid, and docosanoic acid (behenic acid). The contents of hydroxy ricinoleic acid, hydroperoxylinoleic acid and 9-Hydroxy-12-oxo-15(Z)-octadecenoic acid differ significantly between dried and fresh peas. Two other C20 fatty acids, 8, 9-dihydroxy-5,11,14,17-eicosatetraenoic acid and hydroxyicosanoic acid, are significantly higher in dried peas than in fresh peas (Figure 3A).

Amino acids are a type of biological macromolecule in the human body and a substantial basis of biological activity. Lack of any amino acid supply will affect the immune system and other normal functions, making people less healthy and more susceptible to disease [27]. In this study, the contents of all essential amino acids were relatively high in fresh peas. In particular, L-lysine, one of the most important amino acids, which can promote the development and growth of the human body, was much higher in fresh peas than in dried peas (Figure 3B). In addition, except for L-asparagine and L-glycine, the contents of other non-essential amino acids in fresh peas were higher than in dried peas (Figure 3B), Therefore, fresh peas are more beneficial for people in terms of amino acid supplementation.

Sugars provide energy and heat for the body, help maintain the body electrolyte balance, and can be converted into other carbon compounds after ingestion [28]. The common sugars were identified and classified in peas. Visualization of the sugars profile displayed the different changes in their relative contents between dried and fresh peas. As shown in Figure 3C, monosaccharide, disaccharide, trisaccharide and tetrasaccharide were abundant in peas. Moreover, there were also several different kinds of monosaccharides in peas, including tetrose, pentose, hexose and heptose. In addition, the contents of tetrasaccharides and pentasaccharides were high in dried peas, including verbascose, D-maltotetraose, stachyose, and nystose. However, the contents of disaccharides in fresh peas were higher than that in dried peas, except for rutinose (Figure 3C). 

Vitamins, as a large class of chemical compounds, are necessary to maintain complex life activities, but most cannot be synthesized by the human body itself [29]. They are therefore only obtained through food or other fortified products. Here, a total of 17 annotated vitamins were identified (Figure 3D). Isoascorbic acid has the same strong effect of reducibility as ascorbic acid. The isoascorbic acid content was higher in fresh peas than in dried peas, and was not detected in ZW4D. However, 4-pyridoxic acid and 4-pyridoxic acid-o-glucoside were significantly accumulated in dried peas. Other vitamins showed little difference between dried and fresh peas. B-group vitamins have specific effects on brain function, and their deficiency leads to neurological problems [30]. Most importantly, our results also showed that peas are rich in B-group vitamins, including VB2, VB3, VB6, and VB13. In addition, γ-Tocotrienol, the active form of VE, VC and VK1, has important biochemical activities and is abundant in peas (Figure 3D). 

Anti-nutritional factors in foods are responsible for the deleterious effects that are related to the absorption of nutrients that may interfere with the function of certain organs. The presence of alkaloids in foods may induce undesirable effects in humans if their consumption exceeds an upper limit [31]. Previous study showed that high-temperature micronization with infrared rays for the inactivation of alkaloids could increase the nutritional value of legumes for food [32]. Alkaloids, a group of anti-nutritional compounds classified as isoquinoline-type, quinoline-type, indole-type, piperidine-type, pyridine-type and so on, are nitrogen-containing heterocyclic compounds typically isolated from plants [33]. Here, a total of 73 annotated alkaloids were detected, including pyridine alkaloids, plumerane, piperidine alkaloids, and other alkaloids. Observably, the plumeranes accounted for 37% of the total alkaloids, but pyridine alkaloids and piperidine alkaloids only accounted for 7%, respectively (Figure 3E). Interestingly, the content of 6-hydroxynicotinic acid was significantly lower in ZW4F and ZW6F than that in CSRF, which presents the difference between the fresh peas of Zhongwan varieties and the Changshouren variety (Figure 3E). In addition, the presence of abrine—an alkaloid—leads to the toxicity of seeds of *Abrus precatorius* L. (*Gunja*) [34]. The content of abrine in fresh peas was higher than in dried peas (Figure 3E). This provides the basis for us to understand the necessity for eating processed fresh peas, which have more nutritious and healthy value. However, alkaloids also have been shown to be highly biologically active [35], and some show promising pharmacological activity, such as kopsiarborine C and paucidactine C (Figure 3E). Therefore, an in-depth pharmacological evaluation of these compounds should be performed to determine their value for human health. It is significant and valuable for the further development and utilization of peas in future study. 

### 3.4. Identification and Comparative Analysis of the Flavonoid Metabolites in Peas

**Figure 4:**
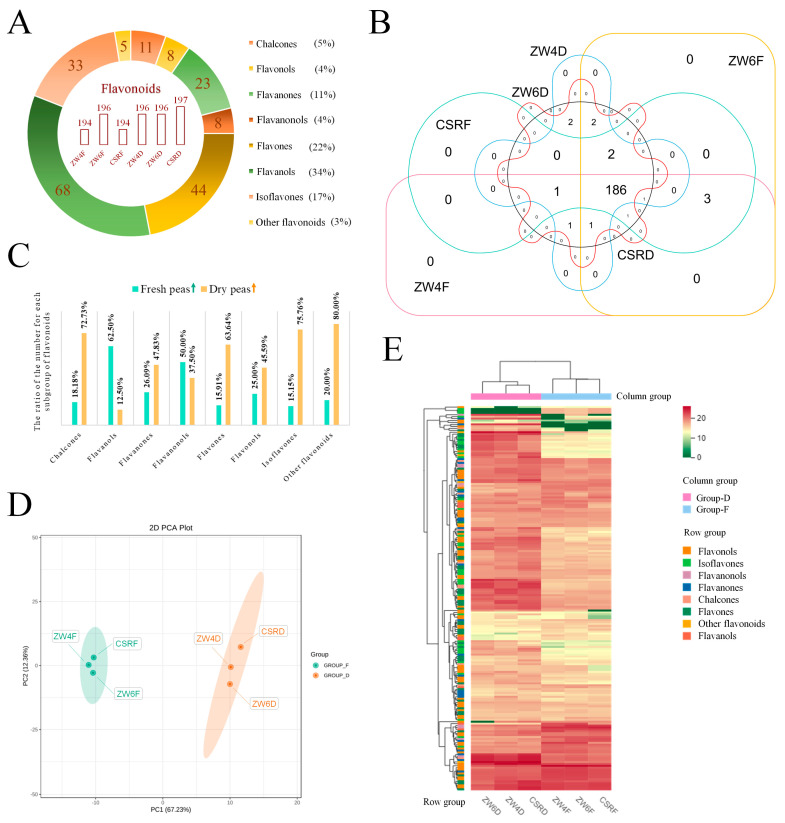
Identification of differential flavonoid metabolites among different peas. (**A**) Classification of a total of 200 flavonoids from three varieties and the number of flavonoids in different fresh and dried peas. (**B**) Venn diagram analysis shows the overlapping and unique flavonoids among different fresh and dried peas. (**C**) The ratio of the number for each subgroup of flavonoids accumulated in all fresh peas or all dried peas from three varieties. (**D**) Principal component analysis of flavonoids from different fresh and dried peas. The PCA score plot of flavonoid metabolites in different peas. (**E**) Cluster heatmap of all flavonoids from different peas. The mean content value of each flavonoid from three biological replicates was normalized by log2 transformation. Columns represent the different peas, and flavonoids are represented by rows. Group-F represents the group of fresh peas, and Group-D represents the group of dried peas.

Flavonoids are present in the combined or free form in many plants such as fruits, vegetables, legumes and tea [36]. The previous study has found that the contents and types of flavonoids in coarse cereals were higher than those in staple food crops [1]. Peas are also one of the important coarse cereals. According to the comparative analysis, 194, 196, 194, 196, 196, and 197 flavonoids were identified from ZW4F, ZW6F, CSRF, ZW4D, ZW6D, and CSRD, respectively, and a total of 200 flavonoids were obtained. The detected flavonoids were divided into eight groups. Flavonols accounted for the largest group (34%), followed by flavones (22%), isoflavones (17%), flavanones (11%), chalcones (5%), flavanols (4%), flavanonols (4%) and other flavonoids (3%) (Figure 4A). From all flavonoids detected,186 were shared in all samples, and none of the other specific flavonoids were identified in any one sample. Particularly, 3′,5,5′,7-Tetrahydroxyflavanone-7-O-glucoside and Kaempferol-3-O-(6′′′′-malonyl) sophorotrioside were only identified in dried peas. Genistein-7-O-galactoside, 4′,5-Dihydroxyisoflavone-7-O-galactoside, and sophoricoside were only contained in fresh peas (Figure 4B). In addition, the contents of 112 flavonoids identified from dried peas were higher than in fresh peas, accounting for 56% of total flavonoids, including 8 chalcones, 1 flavanol, 11 flavanones, 4 flavanonols, 28 flavones, 31 flavonols, 25 isoflavones and 4 other flavonoids (Table S4). The contents of 47 flavonoids were enriched in the fresh seeds of all three varieties, but decreased in dry seeds, including 2 chalcones, 5 flavanols, 6 flavanones, 4 flavanonols, 7 flavones, 17 flavonols, 5 isoflavones and 1 other flavonoid; more detailed information is shown in Table S4. Furthermore, the chalcones, flavones, isoflavones, and other flavonoids accounted for 72.73%, 63.64%, 75.76% and 80.00% of the total number of flavonoids in all dried peas, respectively, but the flavanols accounted for 62.5% of the total number in all fresh peas (Figure 4C, Table S5). The PCA used several principal components to reveal the overall flavonoid differences between multiple variables. Here, two principal components, PC1 and PC2, were extracted and were 67.23% and 12.36%, respectively. The total contribution rate reached 78.59%. For PC1, fresh seeds and dry seeds were clearly separated, and all fresh peas or dried peas from different varieties were packed closely together, indicating a large difference between fresh and dried peas (Figure 4D). This result was consistent with the row clustering of the heatmap (Figure 4E). In addition, the previous study showed that major differences existed in anthocyanin contents for different varieties with different colors of seed coats [37]. However, no anthocyanins were identified here, indicating the yellow or green seed coat color of peas may have no connection with anthocyanin accumulation.

**Figure 5:**
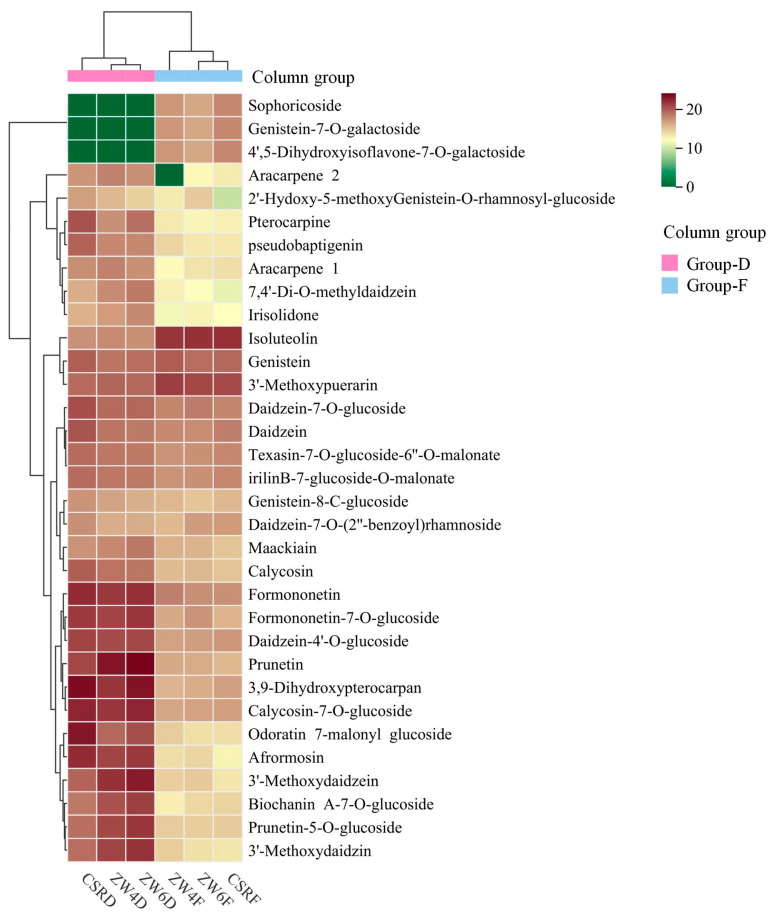
Cluster heatmap of all isoflavones from different peas. The mean content value of each isoflavone from three biological replicates was normalized by log2 transformation. Columns represent the different peas, and isoflavones are represented by rows. Group-F represents the group of fresh peas, and Group-D represents the group of dried peas.

Isoflavones, a subgroup of the flavonoid family, occur naturally in legumes. They show various health benefits often connected with their estrogenic activity and powerful antioxidants, which have been intensively studied for decades [38]. In the early 20th century, isoflavones were found in soybean, but they also exist and are studied in other plants. Daidzein, genistein, glycitein, biochanin A and their glycosylated derivatives are the most common isoflavones found in nature [39]. Daidzein is associated with the control of estrogen, such as in breast cancer, diabetes, osteoporosis, and cardiovascular disease [40]. Genistein is claimed to protect against osteoporosis, reduce the risk of cardiovascular disease, alleviate postmenopausal symptoms and offer anticancer properties [41]. Biochanin A is attracting increasing attention due to its anti-inflammatory, anti-oxidant, anti-cancer and neuroprotective properties [42]. As one of the legumes, we found that peas are rich in isoflavones. A total of 33 isoflavones from peas were detected in this study, and most of them were much richer in dried peas than fresh peas, but sophoricoside, genistein-7-O-galactoside, and 4′,5-dihydroxyisoflavone-7-O-galactoside were accumulated in fresh peas (Figure 5). Moreover, we summarized the kinds and numbers of isoflavones from fruits [43,44], staple food crops [1], coarse cereals [18,45], tuber crops [46,47,48], and vegetables [49,50,51,52,53] in recent reports. They were characterized using the same metabolomic detection platform of MetWare. It was found that the pea has more isoflavones than other plants. Furthermore, isoflavones are relatively more abundant in coarse cereals, and they are relatively few in tuber crops. Based on our study and the summary of previous studies, it was observed that the most common isoflavones are daidzein, genistein and their derivatives in plants (Table 1). Isoflavones showed anticancer, anti-inflammatory, anti-osteoporosis, neuroprotection, and hepatoprotection effects by affecting the different signaling pathways [54]. Thus, our results have instructive and reference value for research on isoflavones in peas.

### 3.5. Putative Metabolic Pathway of Flavonoids in Peas

**Figure 6:**
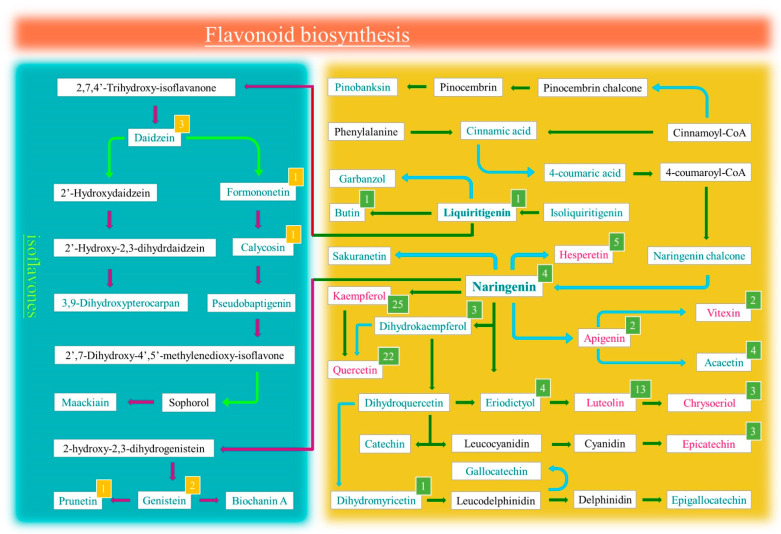
Proposed flavonoid biosynthesis pathway in pea based on KEGG database and this study. Here, only flavonoid metabolites and their glycosylated derivatives are presented, while other derivatives or metabolites whose metabolic locations are not indicated in the KEGG pathway are not shown. Blue letters represent both metabolites and their glycosylated derivatives detected in this study. Pink letters represent that only glycosylated derivatives were detected for a certain compound. Black letters in the red boxes represent the metabolites that were not identified here. The numbers represent the numbers of glycosylated derivatives for a given compound.

Many studies have shown that flavonoids are beneficial to human health, and they have a broad spectrum of biological and pharmacological properties, including antioxidant and anti-inflammatory effects. It is also thought that they have a role in resisting or slowing the formation of tumors [55]. Although different varieties of pea were used in this study, there were almost no differences in the types of flavonoids among them (Figure 4E). Thus, we intended to reveal the pathway of flavonoid metabolism in peas based on the identified flavonoids and their glycosylated derivatives. According to the KEGG database, we preliminarily sorted out the upstream and downstream relationships of different flavonoids. As shown in Figure 6, we used a total of 128 flavonoids to presume the metabolic pathway of flavonoids in this study. Peas are rich in a wide variety of flavonoid derivatives. There are a total of 60 glycosylated derivatives from kaempferol, quercetin and luteolin, which account for 30% of all detected flavonoids. The kaempferol and quercetin belong to flavonols, and luteolin belongs to flavones. The flavonols and flavones were the two largest subgroups of flavonoids in this study (Figure 4A). Kaempferol was the most common flavonoid, and it has been reported that high kaempferol intake reduces the recurrence of advanced colorectal adenomas and reduces the risk of pancreatic adenocarcinoma [37]. Quercetin has molecular and biological functions that render it effective for use as an expectorant, for cough relief, blood lipid reduction, blood pressure reduction, and for cardiovascular disease prevention and treatment [56]. Luteolin has a broad spectrum of anticancer properties and can induce apoptosis of tumor cells mainly by targeting the regulation of signal transduction, gene expression, enzyme activation and inhibition of cell growth [57]. Due to the roles of kaempferol, quercetin and luteolin relevant to human health, peas have very broad application prospects and potential value for exploitation. Obviously, there are two isoflavone synthesis pathways, originating from liquiritigenin and naringenin, respectively (Figure 6). Hence, the results of our study not only give a brief overview of the flavonoid metabolic pathway in peas, but also offer guidance for understanding the utilization value of isoflavones. In addition, it is thought that the pea may possess a broad spectrum of biological and pharmacological properties, such as anti-inflammatory, anti-oxidant and anti-cancer effects. Of course, there are still many flavonoids that need to be further explored and have their metabolic pathways analyzed in follow-up study.

### 3.6. The Guiding Significance of This Study for Future Work

Peas are mainly used as a vegetable, sundry grain and for forage. The nutrient properties and functional composition are therefore neglected in peas. Our research provides important data for people to fully understand the nutrition of peas. We have found that peas are rich in various active ingredients, including some special nutrients, such as B-group vitamins and isoflavones (Figure 3D and Figure 5). Accordingly, we could selectively detect isoflavones or B-group vitamins from many pea germplasm resources and screen pea germplasm with high isoflavone content by quantitative analysis, then use modern breeding techniques to develop new functional pea varieties to meet the nutritional requirements of people in future studies. However, limited by the lack of development of functional genomics for peas, the progress in genetic transformation and molecular biology are far behind those of model crops. Therefore, we urgently need to develop gene-edited peas with modified synthesis pathways for unhealthy compounds, such as abrine (Figure 3E). Finally, new pea-based food products with high nutritive value and that are safe and healthful should be exploited.

## 4. Conclusions

In this study, we applied a widely targeted metabolomics approach to analyze the metabolites of peas. A total of 1095 different annotated metabolites were characterized from different pea varieties, including alkaloids, amino acids and derivatives, flavonoids, lignans and coumarins, lipids, nucleotides and derivatives, organic acids, phenolic acids, terpenoids, and other compounds. We analyzed the differences in the main nutritional compounds and bioactive constituents between dried and fresh peas. It was found that there were a few differences for free fatty acids, vitamins, sugars and alkaloids between dried and fresh peas; however, almost all of the amino acids were highly accumulated in fresh peas. Obviously, abrine—an alkaloid that could lead to the toxicity of seeds—is higher in fresh peas than in dried peas. Analysis also showed that peas are rich in the vitamin B group. Additionally, our study focused on the flavonoid biosynthesis in peas. Comparing the contents and types of flavonoids in all pea samples, we found that a variety of glycosylated derivatives of kaempferol, quercetin, and luteolin are abundant in peas. Moreover, as one of the legumes, peas are richer in isoflavones than many other plants. To sum up, this study provides new insights into the nutrient metabolism of peas and offers a useful reference for the utilization of fresh and dried peas for people to meet their nutritional requirements.

## Figures and Tables

**Table 1:** Detailed information of the isoflavones in peas and other plants from our study and previous studies.

Compound	Pea	Sorghum	Oat	Quinoa	Buckwheat	Millet	Coix	FoxtailMillet	Broomcornmillet	Sorghum	Barley	Rice	Maize	Wheat	Potato	Sweetpotato	Cassavas	CherryTomato	Micro-Tomtomato	Radish	Cucumber	Pepper	Grapes	Red-FleshKiwifruit
2′-Hydroxydaidzein		+	+	+	+	+	+	+	+	+		+	+	+										
2′-Hydroxygenistein		+			+		+		+	+												+		
6-Hydroxydaidzein			+		+		+														+			
Aracarpene2	+										+													
BiochaninA		+	+	+	+	+	+			+			+									+		
Calycosin	+	+		+	+		+									+					+			
Calycosin-7-O-glucoside	+																	+						
Daidzein	+	+	+	+	+	+	+	+	+	+		+	+									+		
Daidzin	+	+	+	+	+																	+		
Formononetin	+	+														+					+			
Genistein	+	+	+		+																	+		
Genistein-7-O-galactoside	+																						+	
Genistein-8-C-glucoside	+										+													
Genistin		+	+	+	+	+	+			+			+					+			+	+	+	
Glycitein		+		+												+					+			
Glycitin		+					+									+								
Ononin	+	+																			+			
Orobol	+	+		+	+		+		+	+														
Prunetin	+	+		+						+			+	+		+						+		
Sissotrin	+			+																	+	+		+
Otherisoflavone	20										1				1			1	2	1	1		5	

Note: “+” represents a compound detected in different plant species.

## Data Availability

The original contributions presented in the study are included in the article; further inquiries can be directed to the corresponding author.

## Supplementary Materials

The following supporting information can be downloaded at: https://www.mdpi.com/article/10.3390/foods13131970/s1, Figure S1. The genetic relationships of Zhongwan varieties used in this study. Figure S2. Correlation analysis of different samples from three pea varieties. Figure S3. Volcano plot of differential metabolites between fresh and dried peas. (A) ZW4F vs. ZW4D, (B) ZW6F vs. ZW6D, (C) CSRF vs. CSRD. Each point in the graph represents a metabolite. The red dots represent upregulated metabolites, the green dots represent downregulated metabolites, and the grey dots represent metabolites with insignificant differences. Figure S4. Cluster heatmap of different metabolites in three comparison groups. (A) Amino acids and derivatives. (B) Lipids. Columns represented the different peas, and the fold-changes of different metabolites in different comparison groups are represented by rows. Figure S5. The top 20 KEGG classifications for the DAMs in the comparisons of ZW4F vs. ZW4D, ZW6F vs. ZW6D, and CSRF vs. CSRD. Table S1. Detailed information of the DAMs in the comparisons of ZW4F vs. ZW4D, ZW6F vs. ZW6D, and CSRF vs. CSRD. Table S2. The numbers of specifically differential metabolites from different subclasses in the three comparison groups. Table S3. The differential accumulated metabolites from each comparison group were annotated by the KEGG database. Table S4. Detailed information of the flavonoids accumulated in all peas from three varieties. Table S5. The numbers of different subgroups of the flavonoids accumulated in all fresh peas or dried peas.
