# Supplementary figures and images for: Widely Targeted Metabolomics Provides New Insights into Nutritional Profiling and Reveals the Flavonoid Pathway of Pea (Pisum sativum L.)
Source: Foods. 2024 Jun 21;13(13):1970. doi: 10.3390/foods13131970 (PMC11240900; doi:10.3390/foods13131970)

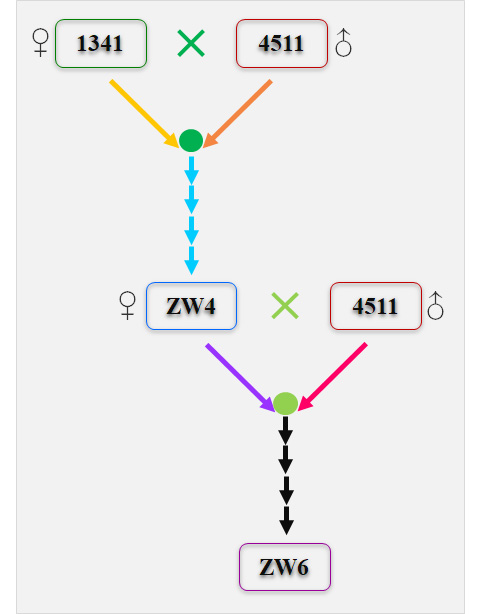

Supplement: Supplementary file 1 [file foods-13-01970-s001.zip › foods-2985415-supplementary/figure-s1.jpg]

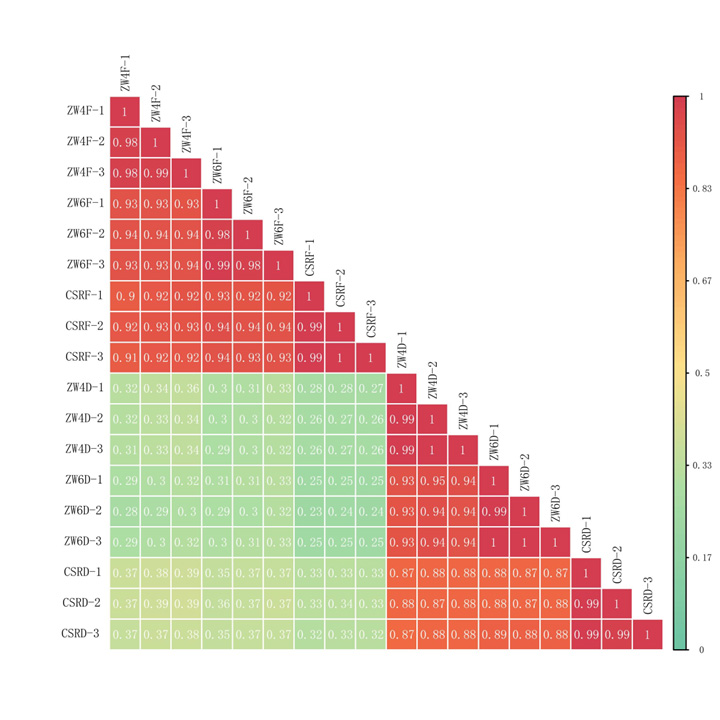

Supplement: Supplementary file 1 [file foods-13-01970-s001.zip › foods-2985415-supplementary/figure-s2.jpg]

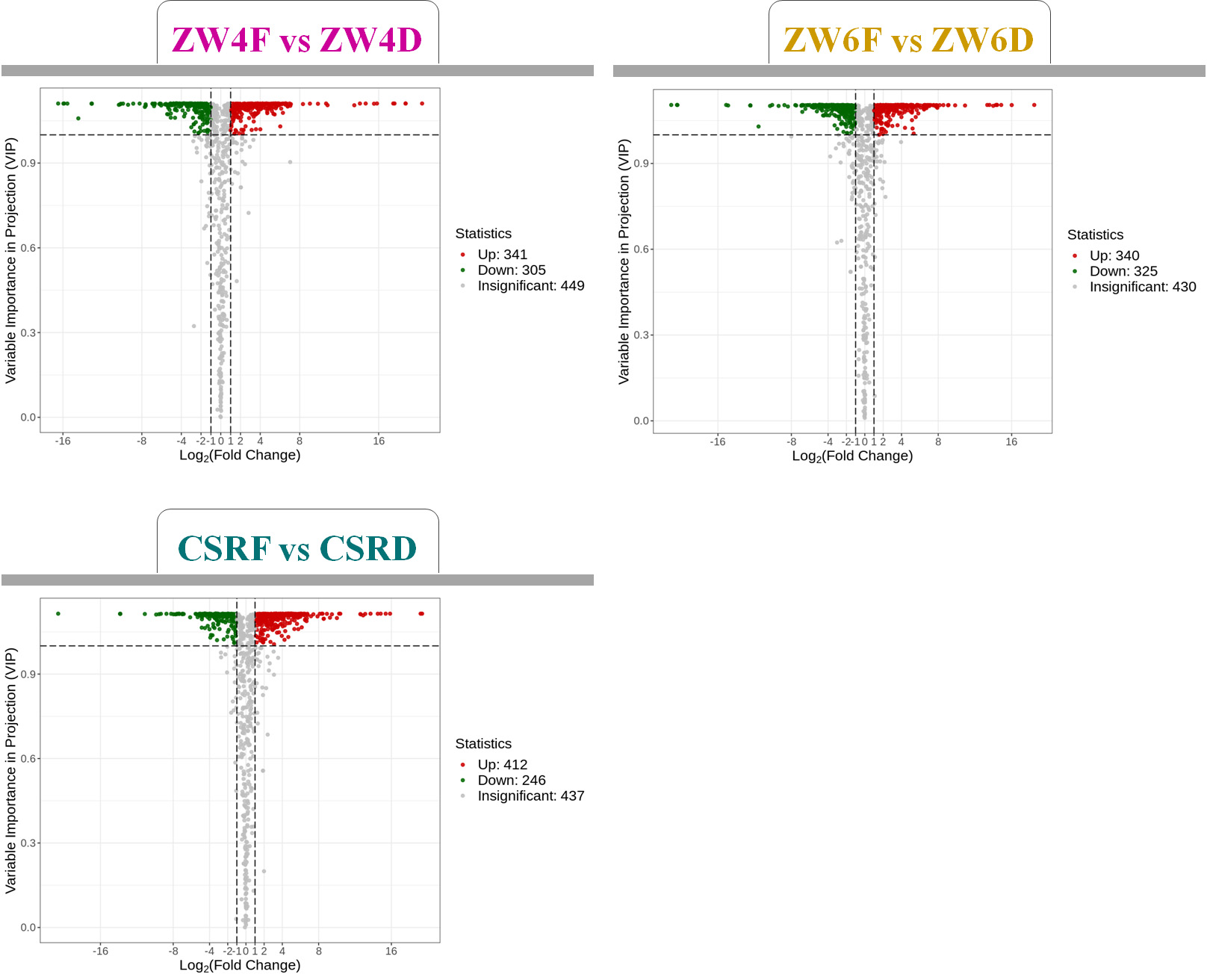

Supplement: Supplementary file 1 [file foods-13-01970-s001.zip › foods-2985415-supplementary/figure-s3.jpg]

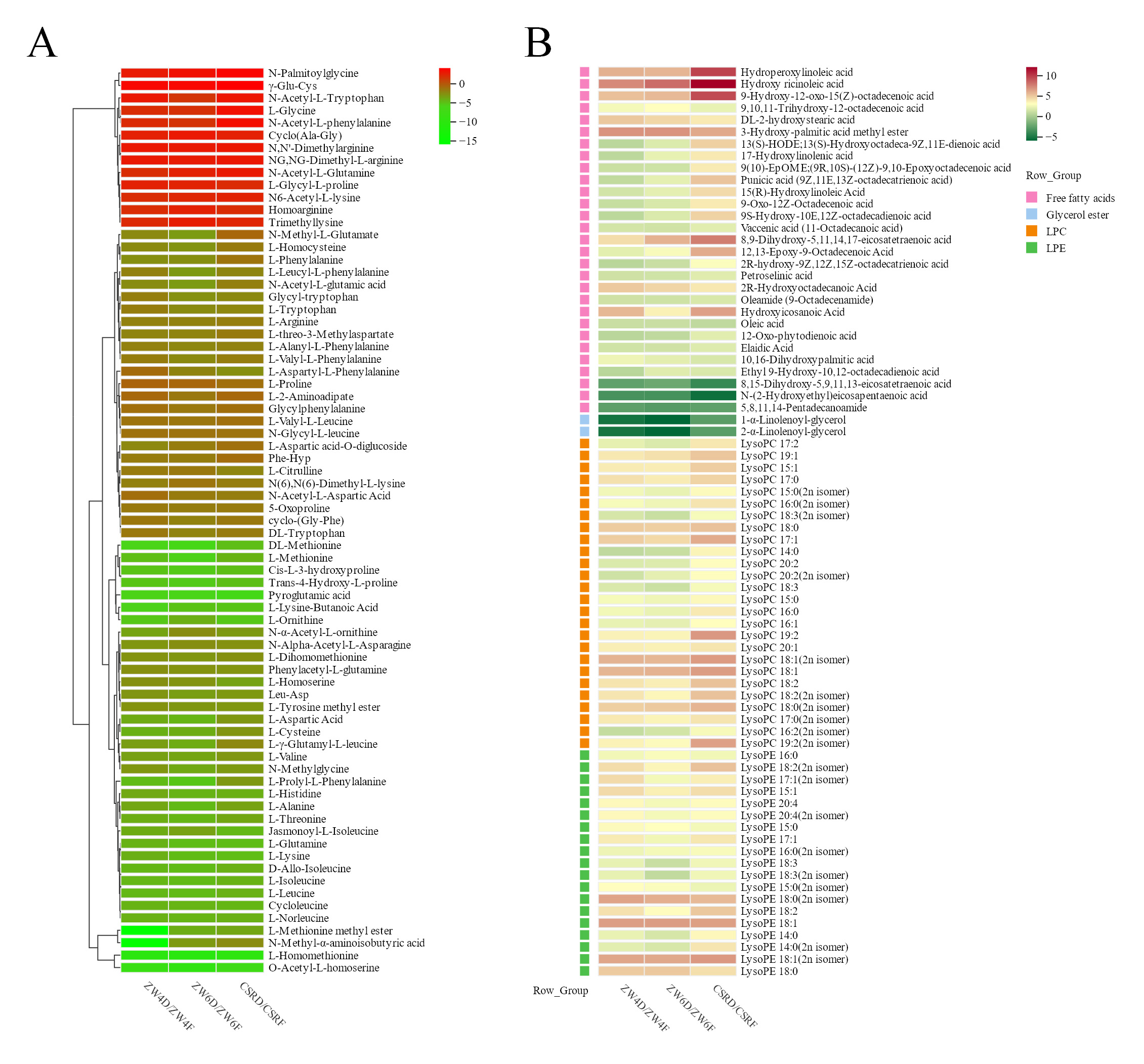

Supplement: Supplementary file 1 [file foods-13-01970-s001.zip › foods-2985415-supplementary/figure-s4.jpg]

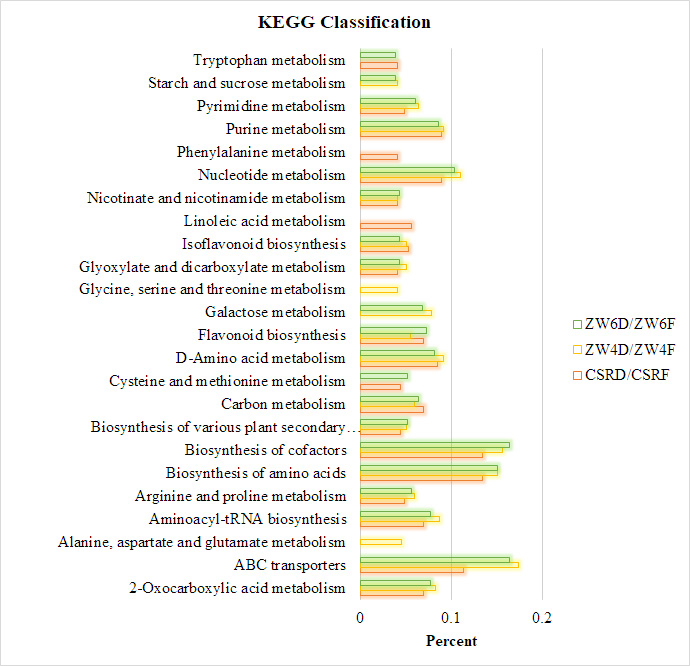

Supplement: Supplementary file 1 [file foods-13-01970-s001.zip › foods-2985415-supplementary/figure-s5.jpg]
